# Si nanowires by a single-step metal-assisted chemical etching process on lithographically defined areas: formation kinetics

**DOI:** 10.1186/1556-276X-6-597

**Published:** 2011-11-16

**Authors:** Androula Galiouna Nassiopoulou, Violetta Gianneta, Charalambos Katsogridakis

**Affiliations:** 1 Institute of Microelectronics (IMEL), National Center for Scientific Research (NCSR) Demokritos, Terma Patriarhou Gregoriou, Aghia Paraskevi, Athens, 15310, Greece

**Keywords:** Si nanowires, metal-assisted chemical etching, lithographically defined areas, formation kinetics

## Abstract

In this paper, we investigate the formation kinetics of Si nanowires [SiNWs] on lithographically defined areas using a single-step metal-assisted chemical etching process in an aqueous HF/AgNO_3 _solution. We show that the etch rate of Si, and consequently, the SiNW length, is much higher on the lithographically defined areas compared with that on the non-patterned areas. A comparative study of the etch rate in the two cases under the same experimental conditions showed that this effect is much more pronounced at the beginning of the etching process. Moreover, it was found that in both cases, the nanowire formation rate is linear with temperature in the range from 20°C to 50°C, with almost the same activation energy, as obtained from an Arrhenius plot (0.37 eV in the case of non-patterned areas, while 0.38 eV in the case of lithographically patterned areas). The higher etch rate on lithographically defined areas is mainly attributed to Si surface modification during the photolithographic process.

**PACS: **68; 68.65-k.

## Introduction

Si nanostructures such as quantum dots, nanocrystals, porous Si, and Si nanowires [SiNWs] exhibit interesting properties [1-4] that are very different from their bulk counterparts and make them interesting for several applications. These properties include a diameter-dependent bandgap, very-high-density electronic states, an increased surface-to-volume ratio, an enhanced exciton binding energy, enhanced thermoelectric properties, and increased surface scattering for electrons and phonons. These properties make SiNWs interesting for application in electronic and photonic devices [4-16], sensors [17], energy harvesting devices, and solar cells [18-20].

Different methods have been developed for the fabrication of SiNWs either by Si etching [12,21,22] or by Si nanowire synthesis [1,23]. Among them, the technique of metal-assisted chemical etching [MACE] [24-29] has gained an increasing interest in the last years due to its simplicity and the high crystalline quality of the obtained SiNWs, resulting from etching of the single crystalline Si material. SiNWs with lengths ranging from a few micrometers to several tens of micrometers can be obtained using either a two-step process involving metal (Ag, Pt) nanoparticle deposition on Si followed by etching or a single-step chemical dissolution process in an aqueous HF solution containing the metal salt. The SiNWs can be fabricated on large areas, which can cover the whole Si wafer. However, for the different applications (Si devices), it is interesting to form the SiNWs on specific confined areas of the Si wafer. It is thus important to develop a technology for their local formation on Si on preselected areas.

In this work, we report on the formation kinetics of SiNWs on lithographically defined areas on the Si wafer using a single-step MACE process based on an aqueous HF/AgNO_3 _solution. We investigated the etch rate of Si, and the corresponding SiNW length, on lithographically defined Si areas compared to that on large non-patterned areas. Field emission scanning electron microscopy [FE-SEM] was used to characterize the samples.

### Experimental details

The substrates used in this work were p-type (100) Si wafers with resistivity ranging from 1 to 2 Ω cm. SiNWs were formed with the single-step MACE technique that consists of immersing the sample in an HF/AgNO_3 _aqueous solution for a process time that determines the SiNW length. With this technique, the mechanism of SiNW formation involves two different processes that occur simultaneously: (a) deposition of Ag nanoparticles on the Si surface and (b) catalytic etching of Si at the sites where the Ag nanoparticles have been deposited. The composition of the AgNO_3_/HF/H_2_O solution used was 0.67 g:35 ml:182 ml. Experiments were carried out in a temperature ranging from 20°C to 55°C. Confined areas on the Si wafer were defined by photolithography using the AZ5214 photoresist. This photoresist was used as the masking material for SiNW formation on confined areas, and it was found to constitute an excellent masking material since it withstands the MACE solution for a long time and it is easily removed with acetone after the end of the process. Square-shaped windows in the photoresist having a surface area ranging from 2 × 2 μm^2 ^to 400 × 400 μm^2 ^were used in our experiments.

SiNWs were characterized by FE-SEM using a JSM-7401F microscope (JEOL, Tokyo, Japan). The Ag dendrite-shaped structures that usually grow on the SiNW surface during the single-step MACE process were removed in an HNO_3_/water solution with a volume ratio of 1:1.

## Results and discussion

### SiNW formation and morphology on large surface areas

In order to understand the experimentally observed differences of the etch rate between large areas and confined areas, it is necessary to recall here the mechanism involved in the one-step MACE process. In this process, the SiNWs are produced by simply immersing the wafer into an HF/AgNO_3 _aqueous solution of a given concentration for an appropriate time. The reaction that takes place is a galvanic displacement reaction. A galvanic cell is established when the Si wafer is immersed into the solution because the reduction potential of the Ag^+^/Ag couple is more positive than the flat band potential of Si. Two simultaneous processes occur in the galvanic displacement reaction at the Si surface: (a) reduction of Ag^+ ^ions by hole injection into Si, which produces metallic Ag deposits (cathodic reaction, electron-consuming type) and (b) oxidation of Si by the injected holes (anodic reaction, electron-releasing or hole-consuming type). In this process, the bonding electrons of surface Si atoms are transferred to Ag^+ ^ions in the aqueous HF solution as described in detail by Peng et al. [26]. The oxidized Si is dissolved by the HF, leading to Si etching and resulting in pore or NW formation. Ag^+ ^reduction and Si oxidation result in the deposition of silver atoms on the cathodic sites of the Si surface, forming nanoscale Ag nuclei at the beginning of the process, with higher electronegativity than Si and thus, strongly attracting electrons from Si to become negatively charged, providing a catalytic surface for further Ag^+ ^reduction. A quasi-Schottky Ag/Si interface is formed, with a relatively low-energy barrier for holes. Charge transfer from the Ag nuclei to Ag^+ ^ions in the solution occurs by hole injection through the quasi-Schottky Ag/Si interface.

**Figure 1:**
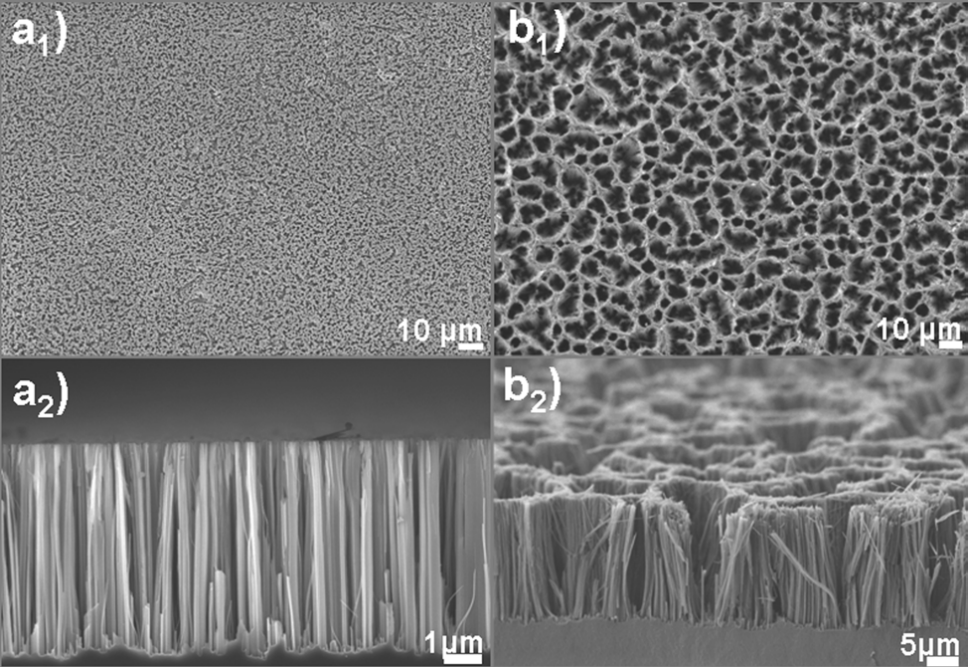
**SEM images of SiNWs of different lengths**. Plane-view (**a_1_**, **b_1_**) and cross-sectional (**a_2_**, **b_2_**) SEM images of SiNWs of two different lengths: 5 μm (a_1_, a_2_) and 20 μm (b_1_, b_2_). It is depicted that short NWs are well separated between them, while longer NWs merge together and form bundles.

Typical scanning electron microscopy [SEM] images of the SiNWs formed by a single-step MACE process are shown in Figure 1: in a_1 _(plane view) and a_2 _(cross section), we see the case of short NWs (5-μm long), which are relatively straight and separated from each other, while in b_1 _(plane view) and b_2 _(cross section), we see the case of longer NWs (20-μm long). It is depicted that after a certain length (a few micrometers), the longer NWs tend to merge together at their tops and form bundles of NWs, as depicted in both the top view (b_1_) and the cross section (b_2_) of Figure 1.

**Figure 2:**
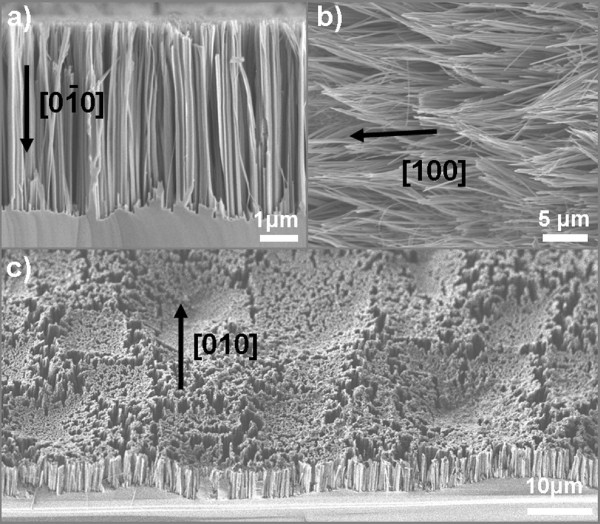
**SEM images of SiNWs grown along the three equivalent crystallographic orientations**. SiNWs on different (100)-equivalent crystallographic orientations: (**a**) front side of the wafer, (**b**) wafer edges, and (**c**) back side of the wafer. The backside wafer surface is rough, and this roughness is reproduced on the SiNW surface since the NW length stays constant all along the surface.

It is worth noting that the etching process by MACE occurs vertically to the Si surface of (100) wafers due to the fact that the reaction rate is much higher in [100] crystallographic orientations than in other orientations. Consequently, when a Si wafer is immersed into the etching solution, SiNWs are formed not only on the front Si wafer surface, but also on the equivalent [100] crystallographic orientations of the sidewalls and the back side of the wafer. In each case, the SiNWs grow perpendicularly to the Si surface. In order to avoid NW formation on the back side of the wafer, it is necessary to cover the backside Si surface with a masking material that prevents Si etching by MACE. The photoresist AZ 5214 was found to be an appropriate choice in this respect. The above effect is illustrated in the SEM images of Figure 2: in (a), we see a cross-sectional SEM image of the front Si surface; in (b), from the sidewalls; and in (c), from the backside surface of the wafer. From this last image, it is depicted that the backside wafer roughness is reproduced at the interface between SiNWs and Si substrate, the NW length being constant and perpendicular to the surface. On the surface of the already formed SiNWs, we do not expect any significant etching due to depletion of the NWs from carriers that occurs during etching.

### Formation of SiNWs on confined areas on the Si substrate

**Figure 3:**
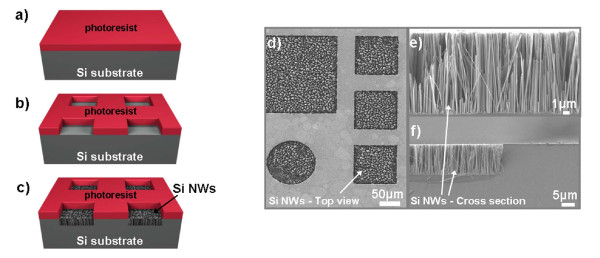
**A schematic representation and SEM images of SiNWs formed on the confined area**. (**a**, **b**, **c**) show the process flow for SiNW formation by MACE on the confined areas, while (**d**) depicts top-view SEM images of the etched confined areas. In (**e**) and (**f**), we see examples of cross-sectional SEM images of the SiNWs. The edge of the etched area is illustrated in (f), depicting that the etching process is relatively anisotropic.

We investigated the growth of SiNWs by MACE on confined areas on the Si substrate of a surface area that varied from 400 × 400 μm^2 ^down to 2 × 2 μm^2^, and we compared the results with those from non-patterned areas. The samples were patterned by photolithography and resist etching. The commercial photoresist AZ 5214 was used as mask for the selective Si etching. The process flow is illustrated in Figures 3a,b,c. The photoresist is deposited on the surface by spinning (a). After exposure to UV light, the exposed resist through the mask is etched away to form Si windows on the resist, which constitute the confined areas to etch by MACE (b). The patterned wafer is then immersed into the MACE solution, and SiNWs are selectively formed in the resist windows, as illustrated in (c). In (d), a top-view SEM image of the SiNWs on the patterned areas is depicted, while in (e) and (f), we see cross-sectional SEM images in two different magnifications of NWs formed in the confined areas at a reaction temperature of 30°C.

The confined surface area ranged from 1 to 4 × 10^4 ^μm^2^. The cross-sectional images are from SiNWs formed in confined areas of 100 × 100 μm^2^, and they are shown at two different magnifications. A first observation is that the NWs formed on confined areas are regular and well defined. They are vertical to the (100) surface, as in the case of NWs formed on non-patterned areas. Their formation at the lithographic edges is relatively anisotropic (see Figure 3f).

**Figure 4:**
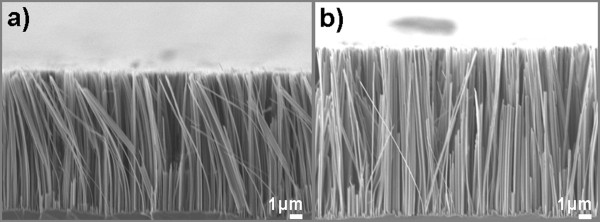
**SEM images of SiNWs formed on the blank Si wafer and on lithographically defined areas**. Cross-sectional SEM images of SiNWs fabricated on non-patterned Si areas (**a**) and on 100 × 100-μm^2 ^lithographically defined areas (**b**).

The most interesting observation is that the SiNW length in the lithographically defined areas is much higher than that in the non-patterned ones, which means that the etching rate is increased in the lithographically patterned surfaces. A direct comparison of the length of SiNWs formed at a temperature of 30°C for an etching time of 1 h is illustrated in the cross-sectional SEM images of Figure 4 for non-confined areas (a) and for lithographically patterned 100 × 100-μm^2 ^areas (b). The difference in length between patterned and non-patterned areas is obvious. For much smaller patterned surfaces (4 × 4 down to 2 × 2 μm^2^), we found that the etching was less regular at the edges.

**Figure 5:**
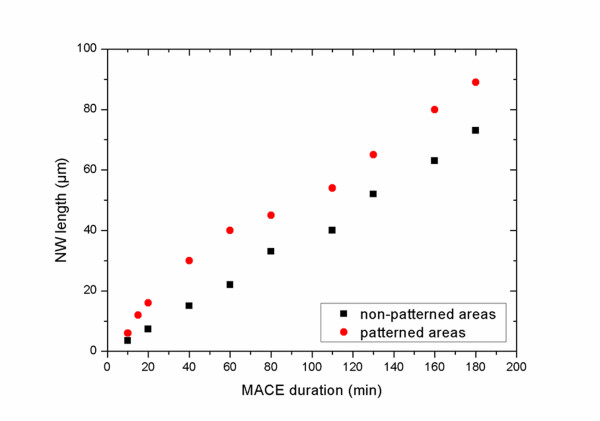
**A graph of the SiNW length versus the processing duration**. The graph shows the evolution of the SiNW length as a function of the etching time by MACE at 30°C of non-patterned areas (black squares) and of 100 × 100-μm^2 ^lithographically defined surface areas (red circles). It is clearly depicted that the etching rate is much higher on lithographically defined areas compared to the non-patterned ones. This mainly occurs at the beginning of the process.

In order to elucidate the above results and understand the mechanism that leads to the different formation kinetics in the two cases, we systematically investigated the etch rate as a function of the etching time of SiNWs formed on non-patterned Si areas and on lithographically defined 100 × 100-μm^2 ^surface areas at a temperature of 30°C. The results are shown in Figure 5. In non-patterned areas, the SiNW length increases linearly with the etching time, in agreement with results in the literature [30]. On the other hand, on lithographically patterned areas, the etch rate is higher at the beginning of the process and becomes almost parallel to the etch rate of non-patterned areas after an etching time of approximately 60 min. The etch rate of large non-patterned areas was found to be approximately 0.4 μm/min, while for the 100 × 100-μm^2 ^areas, it was 0.66 μm/min for an etching time from 0 to 60 min and 0.45 μm/min at higher etching times.

**Figure 6:**
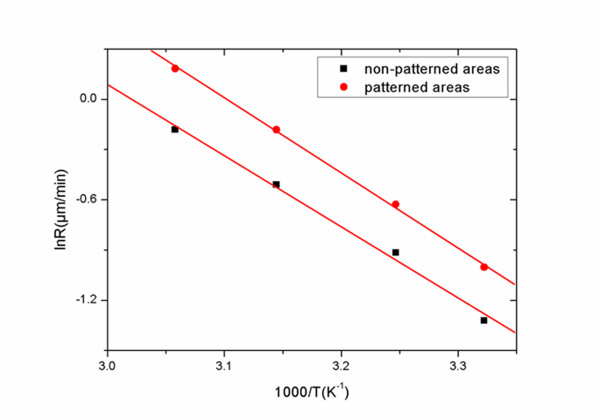
**Arrhenius plots of the formation rate of SiNWs on non-patterned and on lithographically defined areas**. Arrhenius plots of the etch rate of Si by MACE as a function of temperature in the case of non-patterned areas (dark squares) and in the case of lithographically defined confined areas (red circles). From these plots, the process activation energy is calculated at approximately 0.37 eV in the first case and at approximately 0.38 eV in the second case.

We also investigated the temperature dependence of the Si NW length in a temperature ranging from 20°C to 50°C. The SiNW formation rate was found to be linear with temperature in both cases, with an activation energy derived from an Arrhenius plot and is equal to 0.37 eV in the case of non-patterned areas (in good agreement with Cheng et al. [30]) compared to 0.38 eV in the case of lithographically patterned areas. The two Arrhenius plots are almost parallel to each other, showing that the thermal activation process is the same in both cases (see Figure 6).

In order to understand the mechanism responsible for the increase of the formation rate of Si NWs on lithographically patterned areas compared to the non-patterned ones, we performed a series of experiments and examined different factors that could be at the origin of the different reaction kinetics in the two cases, as follows:

#### Effect of surface area

In order to elucidate the effect of surface area localization on the formation rate of SiNWs, we performed a series of experiments on a single wafer with different lithographically defined surface areas. The wafer was immersed into the solution for 30 min at a temperature of 30°C for a reaction time of 1 h. A comparative study of cross-sectional SEM images of the SiNWs from the different surface areas of the sample showed that there was no significant difference in the SiNW length in small and large confined surface areas in the examined range from 50 × 50 μm^2 ^to 400 × 400 μm^2^.

#### Effect of silver dendrite density

**Figure 7:**
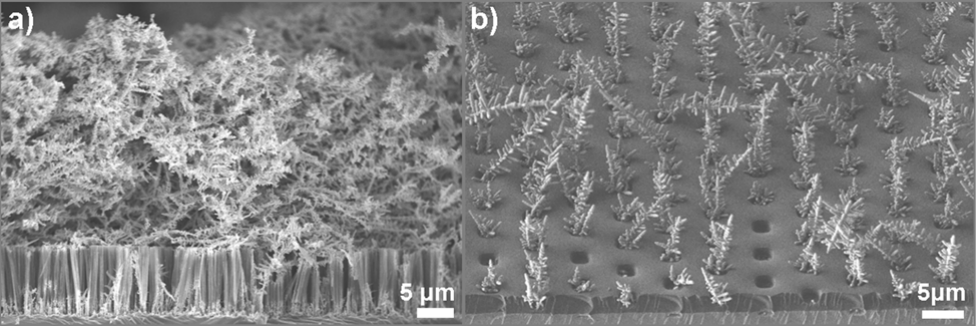
**SEM images of the samples right after the MACE process and before the Ag removal**. Dendrite Ag nanostructures on the SiNW surface during the MACE process on non-patterned areas (**a**) and on lithographically defined 2 × 2-μm^2 ^surface areas (**b**).

During the MACE process, Ag is initially deposited on the Si surface, and a galvanic cell is established when Si etching is initiated; Ag nanoparticles proceed into the Si substrate through Si etching. These particles are found at the interface of the etched area with the Si substrate. On the other hand, silver continues to be deposited on the Si surface, forming dendrite nanostructures, which are very dense in the case of large non-confined areas. In the case of confined areas, the dendrite nanostructures formed on the Si surface are localized on Si areas not covered by the photoresist. They grow locally and are less dense, thus allowing for an easier interaction of the Si surface with the metal ions from the chemical solution. This is illustrated in Figure 7, where we see tilted cross-sectional SEM images from a non-patterned (a) and a 2 × 2-μm^2 ^lithographically defined surface area (b). In (a), the dendrites are very dense and completely cover the etched area, while in (b), they protrude from the small etched areas, leaving more free spaces between them, thus facilitating the interaction of Si with the chemical solution. We performed experiments by changing the inter-distance between small etched areas, and we found again that there was no significant difference between confined areas with different inter-area distances between them. This rules out the hypothesis that the dendrite density could be at the origin of the difference in the etching rate by MACE between the confined and non-confined areas.

#### Effect of surface modification through the lithographic steps

After excluding all the factors above, we examined the possible effect of the lithographic steps on the reaction kinetics by MACE in the case of lithographically defined areas. The different process steps include the use of a resist adhesion promoter, resist spinning, UV exposure, and a developer for resist stripping. The first experiment was to perform all the lithographic steps above on a blank wafer without any mask exposure, remove the resist normally, and then, etch the sample by MACE. In this way, we exclude the surface area effect from the origin of the etch rate difference. Indeed, after applying all lithographic steps without mask and stripping the resist, the etch rate of the sample was much higher than the etch rate of a virgin Si wafer. This leads to the conclusion that surface modification during lithography is at the origin of the etch rate effect described above. The step responsible for surface modification is the adhesion promoter. The one used was hexamethyldisilzane [HMDS], known to remove -OH groups from the Si surface and form a hydrophobic surface with the methyl groups of the HMDS fragment. The so-formed hydrophobic surface improves resist wetting and adhesion. We demonstrated that it also increases the etch rate by MACE.

After the above result, we investigated a number of other surface chemical treatments on the MACE kinetics. We compared the SiNW length after etching by MACE at 28°C for 1 h on a non-treated Si wafer, a Si die sample after piranha cleaning for 30 min, and a Si die after HF (10%) dip for 15 min and after a dip into the above-mentioned developer for approximately 1 min. The result was that the non-treated wafer resulted in 20-μm-long NWs; the same was obtained after piranha cleaning, while there was approximately 15% increase in length after the developer dip and 7.5% decrease in length after the HF dip.

From the above experiments, it is clear that chemical surface modification plays an important role in the etching kinetics by MACE. It mainly affects these kinetics at the beginning of the process.

## Conclusion

A comparative study on the formation kinetics of SiNW formation by MACE of non-patterned and lithographically patterned Si surfaces showed that the etch rate of Si for SiNW formation is higher in the case of lithographically patterned surfaces compared to that of the non-patterned ones. The origin of this effect is at the surface modification by the promoter used for resist adhesion. Resist promoter is known to form a hydrophobic surface covered by methyl groups. Moreover, it was shown that surface treatment by an HF dip, which leads to surface passivation by hydrogen, results in retardation of the etching process, thus leading to shorter SiNWs.

## Competing interests

The authors declare that they have no competing interests.

## Authors' contributions

AGN supervised all the work and wrote the paper, VG helped in developing the Si nanowire etching process and performed all the experiments related to nanowire growth on patterned surfaces, while HK was involved in the initial experiments on Si etching by MACE. All authors read and approved the final manuscript.
